# A Rare but Real Necessity: Case Report of Coronary Artery Stenting in an Infant

**DOI:** 10.1155/2022/3815465

**Published:** 2022-02-22

**Authors:** José D. Martins, Guilherme Lourenço, Lídia Sousa, Fátima F. Pinto

**Affiliations:** ^1^Pediatric Cardiology Department, Hospital de Santa Marta, Centro Hospitalar de Lisboa Central EPE, Lisbon, Portugal; ^2^Cardiology Department, Hospital de Santa Marta, Centro Hospitalar de Lisboa Central EPE, Lisbon, Portugal

## Abstract

Percutaneous coronary intervention (PCI) is an extremely common and well-established procedure in adults which is rarely performed in children. We present a case of a successful left main coronary artery stenting in a small infant with a congenital coronary artery anomaly. We highlight the technical challenges of performing a PCI in a small patient, the risks of antithrombotic prophylaxis in this age group, and the importance of the combined work of the adult and pediatric interventional cardiologist.

## 1. Introduction

Percutaneous coronary intervention (PCI) is a very common procedure in adults, but extremely rare in children. In the latter, coronary artery stenosis requiring intervention is exceptional, particularly in infancy, where we found only seven such cases [1–6]. Older children or adolescents may also present with coronary compromise requiring PCI, such as after percutaneous pulmonary valve implantation [7], Kawasaki disease [8], or heart transplantation [9].

Congenital coronary anomalies are sporadic findings that occur either as an isolated anomaly of the coronary origin or course (such as ALCAPA) or in association with other heart anomalies (such as transposition of the great arteries). These patients are managed surgically, usually with coronary reimplantation. The incidence of coronary artery stenosis requiring PCI in infancy occurs only as a rare complication after these coronary artery surgeries [1–6].

We present a successful case of coronary artery stenting in an infant with ALCAPA with a significant postoperative stenosis.

## 2. Case Report

Our patient was born overseas, in São Tomé and Principe, a small island nation off the western coast of Africa. Despite early symptoms of overt heart failure, she was only evacuated to our country at the age of 18 months, weighing 8.9 kg. Upon arrival, we made the echocardiographic-based diagnosis of an anomalous left coronary artery (LCA) from the pulmonary artery (ALCAPA) with a dilated and poorly functioning left ventricle (LV). The patient underwent uneventful left coronary artery implantation in the left-facing aortic sinus. Due to persistent postoperative hemorrhage around the site of LCA reimplantation, biological glue was used, with success. However, in the postoperatory period, there was no improvement of the LV function and size, and the patient remained in low cardiac output. The CT scan showed a significant left main coronary stenosis and ruled out intracoronary thrombosis.

**Figure 1:**
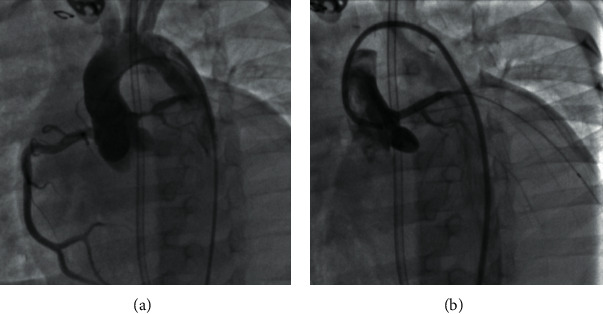
On the left-hand-sided panel (a), the angiography reveals a significant stenosis of the left main coronary artery. The right-hand-sided panel (b) depicts an excellent angiographic result after stent implantation in the left main coronary artery, which is now widely patent.

On the 4^th^ postoperative day, the patient underwent cardiac catheterization (Figure 1). It ruled out left coronary artery ostial narrowing at the anastomosis site but showed a severe rosary-bead-like stenosis (minimum diameter 1.1 mm) that involved the whole left main coronary artery and extended into the ostia of the left anterior descending and circumflex arteries. The heart team discussed the case on-site, and the patient was refused for surgical reintervention, and therefore, a PCI was attempted. Informed consent was obtained prior to the procedure.

We were unable to selectively catheterize the left coronary artery with several guiding catheters, which proved too large for the patient size. Therefore, we used a 4Fr diagnostic Judkins left 2.5 catheter to catheterize the left coronary artery with a 0.014^″^ hydrophilic coronary guidewire, a Runthrough NS Hypercoat (Terumo Interventional Systems, Somerset, NJ, USA), which was placed distally in a diagonal artery. We exchanged over-the-wire the diagnostic catheter for a 5Fr Judkins left 3.5 guiding catheter, which was left in the left-sided sinus of Valsalva. The coronary stenosis was inframillimetric, and the normal anterior descending coronary artery measured 3 mm. We implanted a 3 × 12 mm Xience Sierra everolimus drug-eluting coronary stent system (Abbott Vascular, Santa Clara, CA, USA). The immediate postinterventional coronarography showed an optimal angiographic result, with adequate distal flow to both the anterior descending and circumflex coronary arteries.

The patient was placed under dual antiplatelet therapy with aspirin (5 mg/kg/day) and clopidogrel (0.2 mg/kg/d). Despite a widely patent flow in the coronary stent, well seen in serial transthoracic echocardiograms, the left ventricle function and size did not recover. The patient required a left-ventricular assist device due to persistent low cardiac output syndrome. She eventually died, seven days later, with sepsis.

## 3. Discussion

Postoperative LCA occlusion and persistent LV dysfunction after ALCAPA surgery are not uncommon [10, 11]. In our case, we speculate that external compression (due to the biological glue, blood clots, or other mechanisms) or coronary artery kinking due to surgical proximal segment mobilization may have caused the LCA stenosis. Another contributing factor could be the presence of intracoronary (so-called “steal”) collaterals that may exist in ALCAPA and result in competitive flow to the normal coronary artery system [12].

We are aware that coronary artery stenting in this situation is only tolerable as a bailout technique in a critically ill infant, especially considering the risks of stent thrombosis and dual antiplatelet antiaggregation in a small infant and the fixed stent diameter which will not accompany the patient's somatic growth.

There are many challenges associated with coronary artery stenting in infants which make this a high-risk procedure. All seven published case reports were technically successful but carried significant acute and short-term complications, including myocardial ischemia, need for extracorporeal membrane oxygenator support, early in-stent thrombosis, and premature death. Several factors contributed to these findings, including the adverse baseline clinical situation that leads to the cardiac catheterization, the small patient weight, the diminutive coronary diameter, and the multiple anatomic variants in a postoperative situation. Additionally, there is a limited availability of adequate coronary catheterization hardware, which is designed for adult-sized coronary arteries and vessels, that can be cumbersome to manage in small patients. The choice of bare metal stent (BMS) vs. drug-eluting stent (DES) is also up for debate, due to concerns about potential deleterious side effects of systemic circulation of antiproliferative drugs in small patients [13]. In the previous reports, only 2/7 stents were DES, the remainder were BMS. Considering preliminary safety data of neonatal DES in the ductus arteriosus, we opted for a recent generation of DES. There is also debate about the optimal thromboprophylaxis regimen, which was dual-antiplatelet therapy in 5/7 and single-antiplatelet (aspirin) in the remainder. We chose a dual-antiplatelet regimen, which is the recommended thromboprophylaxis for DES. We had no hemorrhagic complications. Finally, there is a challenge due to the understandable lack of coronary artery expertise of the pediatric interventionalists. In our case, it was crucial to perform a four-hand procedure with an experienced adult congenital heart disease interventionalist.

Our case highlights the importance of collaborative work between adult and pediatric cardiologists that allows a patient-focused treatment and some technical challenges of this challenging interventional technique in infants.

## Supplementary Materials

Supplementary Materials Supplementary Material (video legend): available at: https://drive.google.com/drive/folders/10W_b_ZjXjJS7ui5uhers6xJrQiGC_uc3?usp=sharing. Part 1: preintervention aortic root angiography, showing a right dominant system and a severe stenosis of the left main coronary artery. Part 2: 4Fr diagnostic Judkins left 2.5 catheter is used to catheterize the left coronary artery with a 0.014^″^ hydrophilic coronary guidewire. Part 3: preintervention selective left coronary artery angiography, showing a severe rosary-bead-like stenosis that involved the whole left main coronary artery and extended into the ostia of the left anterior descending and circumflex arteries. Parts 4 and 5: postintervention selective angiographies, showing an adequately placed stent in the left main coronary artery, with a good angiographic result.

## References

[1] Bratincsak A., Salkini A., El-Said H. G., Moore J. W. (2012). Percutaneous stent implantation into coronary arteries in infants. *Catheterization and Cardiovascular Interventions*.

[2] Hallbergson A., Rome J. J. (2015). Percutaneous left main coronary artery stent for acute myocardial ischemia after repaired ALCAPA. *Catheterization and Cardiovascular Interventions*.

[3] Schneider M., Wiebe W., Hraska V., Zartner P. (2013). Coronary interventions in congenital heart diseases: from preterm to young adult patients. *Journal of Interventional Cardiology*.

[4] de Lezo J. S., Pan M., Herrera C. (2009). Combined percutaneous revascularization and cell therapy after failed repair of anomalous origin of left coronary artery from pulmonary artery. *Catheterization and Cardiovascular Interventions*.

[5] Chrysant G. S., Balzer D., Taniuchi M. (2005). Left main stem coronary artery stenting in a 3-month-old child after anomalous left coronary artery from pulmonary artery repair. *Pediatric Cardiology*.

[6] Schneider A. E., Johnson J. N., Taggart N. W. (2014). Percutaneous coronary intervention in pediatric and adolescent patients. *Congenital Heart Disease*.

[7] Divekar A. A., Lee J. J., Tymchak W. J., Rutledge J. M. (2006). Percutaneous coronary intervention for extrinsic coronary compression after pulmonary valve replacement. *Catheterization and Cardiovascular Interventions*.

[8] Muta H., Ishii M. (2010). Percutaneous coronary intervention versus coronary artery bypass grafting for stenotic lesions after Kawasaki disease. *The Journal of Pediatrics*.

[9] Shaddy R. E., Revenaugh J. A., Orsmond G. S., Tani L. Y. (2000). Coronary interventional procedures in pediatric heart transplant recipients with cardiac allograft vasculopathy. *The American Journal of Cardiology*.

[10] Secinaro A., Ntsinjana H., Tann O. (2011). Cardiovascular magnetic resonance findings in repaired anomalous left coronary artery to pulmonary artery connection (ALCAPA). *Journal of Cardiovascular Magnetic Resonance*.

[11] Castaldi B., Vida V., Reffo E. (2017). Speckle tracking in ALCAPA patients after surgical repair as predictor of residual coronary disease. *Pediatric Cardiology*.

[12] Frommelt M. A., Miller E., Williamson J., Bergstrom S. (2002). Detection of septal coronary collaterals by color flow Doppler mapping is a marker for anomalous origin of a coronary artery from the pulmonary artery. *Journal of the American Society of Echocardiography*.

[13] Aggarwal V., Dhillon G. S., Penny D. J., Gowda S. T., Qureshi A. M. (2019). Drug-eluting stents compared with bare metal stents for stenting the ductus arteriosus in infants with ductal-dependent pulmonary blood flow. *The American Journal of Cardiology*.

