# Supplementary material for: A Rare but Real Necessity: Case Report of Coronary Artery Stenting in an Infant
Source: Case Rep Cardiol. 2022 Feb 22;2022:3815465. doi: 10.1155/2022/3815465 (PMC8888074; doi:10.1155/2022/3815465)
Supplement: Supplementary Materials — Supplementary Material (video legend): available at: https://drive.google.com/drive/folders/10W_b_ZjXjJS7ui5uhers6xJrQiGC_uc3?usp=sharing. Part 1: preintervention aortic root angiography, showing a right dominant system and a severe stenosis of the left main coronary artery. Part 2: 4Fr diagnostic Judkins left 2.5 catheter is used to catheterize the left coronary artery with a 0.014″ hydrophilic coronary guidewire. Part 3: preintervention selective left coronary artery angiography, showing a severe rosary-bead-like stenosis that involved the whole left main coronary artery and extended into the ostia of the left anterior descending and circumflex arteries. Parts 4 and 5: postintervention selective angiographies, showing an adequately placed stent in the left main coronary artery, with a good angiographic result. [file 3815465.f1.docx]

**SUPPLEMENTARY MATERIAL (VIDEO LEGEND):**

Available at:

<https://www.dropbox.com/sh/1843wl5xikkjv9a/AACtBOfjK0y0SFFrxnv0gPRNa?dl=0>

Part 1: Pre-intervention aortic root angiography, showing a right dominant system and a severe stenosis of the left main coronary artery

Part 2: 4Fr diagnostic Judkins left 2.5 catheter is used to catheterize the left coronary artery with a 0.014’’ a hydrophilic coronary guidewire

Part 3: Pre-intervention selective left coronary artery angiography, showing a severe rosary-bead like stenosis that involved the whole left main coronary artery and extended into the ostia of the left anterior descending and circumflex arteries

Parts 4 and 5: Post-intervention selective angiographies, showing an adequately placed stent in the left main coronary artery, with a good angiographic result
